# Low circulating 25-hydroxyvitamin D level is associated with increased colorectal cancer mortality: a systematic review and dose–response meta-analysis

**DOI:** 10.1042/BSR20201008

**Published:** 2020-07-29

**Authors:** Guanghai Wu, Mei Xue, Yongjie Zhao, Youkui Han, Shuai Zhang, Judong Zhang, Chao Li, Jing Xu

**Affiliations:** 1 Department of General Surgery, Tianjin Union Medical Center, Jieyuan Road 190, Hongqiao District, Tianjin, 300121, P.R. China; 2 NHC Key Laboratory of Hormones and Development, Tianjin Key Laboratory of Metabolic Diseases, Chu Hsien-I Memorial Hospital and Tianjin Institute of Endocrinology, Tianjin Medical University, Tianjin 300134, P.R. China

**Keywords:** colorectal cancer, meta analysis, prognosis, vitamins

## Abstract

Epidemiological studies have suggested inconclusive associations between 25-hydroxyvitamin D (25(OH)D) and survival in patients with colorectal cancer (CRC). The aim of the present study was to quantitatively assess these associations. PubMed, EMBASE, and Web of Science databases were systematically searched for eligible studies. Subgroup analyses based on study geographic location, publication year, length of follow-up time, sample size, and stage were conducted to explore the potential sources of heterogeneity. Dose–response relationships and pooled hazard ratios (HR) for overall and CRC-specific survival comparing the highest versus the lowest categories of circulating 25(OH)D concentrations were assessed. Overall, 17 original studies with a total of 17,770 CRC patients were included. Pooled HR (95% confidence intervals) comparing highest versus lowest categories were 0.64 (0.55–0.72) and 0.65 (0.56–0.73) for overall and CRC-specific survival, respectively. Studies conducted in the U.S.A., with median follow-up time ≥ 8 years, larger sample size, and including stage I-III patients showed a more prominent association between 25(OH)D concentrations and overall survival. The dose–response analysis showed that the risk of all-cause mortality was reduced by 7% (HR = 0.93; 95% CI: 0.90, 0.95), and the risk of CRC-specific mortality was reduced by 12% (HR = 0.88; 95% CI: 0.84, 0.93) for each 20 nmol/l increment of 25(OH)D concentration. This meta-analysis provides evidences that a higher 25(OH)D concentration is associated with lower overall mortality and CRC-specific mortality.

## Introduction

Colorectal cancer (CRC) is one of the most common cancers and the second cause of cancer-related mortality in the world [1]. It is estimated that, over 1.8 million new cases are clinically diagnosed with CRC, and approximately 881,000 deaths due to CRC are estimated to occur worldwide in 2018 [1]. Currently, the traditional therapies, such as chemotherapy [2], surgical resection [3], radiotherapy [4], and combined therapy [5] are used for CRC treatment. Over the past decade, many clinical or experimental studies have provided many fundamental insights into the pathogenesis of CRC [5–7]. There are a number of risk factors for CRC reported in published studies, such as diet [8], obesity [9], and alcohol intake [10]. However, there are still a limited number of modifiable risk factors identified for CRC.

Vitamin D is a steroid hormone known to play a role in calcium balance and skeletal physiology. The roles of vitamin D in human diseases have received increased attention, and it has been regarded as a vital hormone to maintain the normal functions of various organs or systems in the bodies [11]. Epidemiologic studies have reported the association between vitamin D and key adverse health outcomes, such as cardiovascular and cancer-related morbidity and mortality [12]. Studies have suggested that vitamin D has the properties of anti-inflammation [13], anti-oxidative stress [14], reducing cell proliferation [15], and regulating cellular differentiation [16] in various cancers cells. As early as 1980, an epidemiological study proposed that vitamin D may have anti-cancer properties [17]. Since then, numerous literatures supported this hypothesis [18–20]. *In vitro* studies and research using animal models, mostly used 1,25(OH)2D as intervention, have suggested that vitamin D might slow or prevent the development of cancer, through regulating cell proliferation, apoptosis, and promoting cellular differentiation [16,21].

Owing to the tight homeostatic regulation of the production and levels of 1,25(OH)2D and the short half-life, studies in general used circulating 25-hydroxyvitamin D (25(OH)D) to reflect vitamin D status [22]. Serum levels of 25(OH)D, a precursor of activated vitamin D, increase in response to exposure to sunlight, vitamin D-rich diet, or vitamin D supplementation [11]. Cancer cells are believed to take up and activate 25(OH)D within the cell, which binds to the vitamin D receptor to regulate gene expression and consequently regulating the pathophysiology of tumor [23]. Since more than 30 years ago, the first clinical study reported an inverse association between 25(OH)D and CRC was published [24]. However, the results from prospective cohort studies have been inconsistent. In several cohort studies, higher 25(OH)D levels were associated with lower total cancer incidence and lower total cancer mortality in colorectal cancer, while there were also studies reported null association [25–27] or U-shaped association [28].

At present, most literatures focused on the relationship between vitamin D and the risk of CRC. Although several meta-analysis of prospective studies have summarized the association between blood 25(OH)D concentrations and survival in patients with CRC [29,30]. However, due to the small numbers of studies and patients included. The previous reviews could not fully explore the potential variation of this association. Therefore, to provide a more comprehensive, up-to-date assessment of the associations between 25(OH)D and CRC survival, we conducted a meta-analysis to quantitatively assess these associations, with a particular focus on associations within subgroups. In addition, a dose–response meta-analysis was also performed to explore the trend estimation.

## Materials and methods

### Search strategy

We searched PubMed, EMBASE databases and Web of science from inception to March 1, 2020, for eligible studies on the relationship between vitamin D and mortality of CRC patients. The terms used to retrieve literatures were the following: CRC OR colorectal cancer OR colorectal tumor OR colorectal neoplasms OR colon cancer OR rectal cancer AND vitamin D OR 25(OH)D OR 25-hydroxyvitamin D AND prognosis OR mortality OR survival. We also referred to the reference lists from reviews or relevant papers to get more eligible researches. Conference abstracts were also included if sufficient data was provided. There was no language restriction. Two authors independently performed the literature search and identified potential studies of the title, abstract and full-text.

### Selection criteria

Reports were included if they met the criteria as follows: (1) the association between vitamin D and mortality in CRC was reported; (2) study designs: case–control studies, cohort studies and randomized control trials (RCTs); (3) the risk estimates of mortality in CRC, like hazard ratios (HR) and 95% confidence interval (CI) for overall survival (OS) or CRC-specific survival (CSS) were reported. If the same data were used in several studies, we selected the publication with the largest number of cases or more details. The exclusion criteria: (1) letters, editorials, abstracts, reviews, case reports or expert opinions; (2) studies not based on people; (3) outdated articles with little significance or credibility.

### Data extraction

From each included study, data were independently extracted by two investigators (Guanghai Wu and Jing Xu) using a standardized data extraction form. Briefly, we recorded study characteristics including first author name, publication year, country and mean/median duration of follow-up. We also extracted the following information about study populations: sample size, sex, CRC stage, age, and number of total and CRC death. In addition, we extracted data about the categories of blood 25(OH)D concentrations and the median/mid-point/interval of 25(OH)D concentrations in each category. Finally, we recorded HR and 95% CIs for the association of 25(OH)D with OS and CSS. Disagreements between investigators were discussed and resolved by an additional reviewer (Yongjie Zhao). In this report, blood 25(OH)D concentrations were expressed in nmol/l. To convert concentrations reported in ng/ml, an adequate conversion factor (1 ng/ml = 2.5 nmol/l) was used.

### Quality assessment

Quality of included studies was evaluated by use of the Newcastle–Ottawa Scale (NOS). According to its criteria, studies were assessed on the basis of three perspectives: selection, comparability, and outcomes. If studies got seven or more stars, they regarded as high quality. Differences were resolved by discussion.

### Statistical analysis

The pooled HR and 95% CI for the association of categories of patients with highest versus lowest 25(OH)D concentrations with overall and CRC-specific survival were estimated. Statistical heterogeneity among studies was evaluated with the use of Cochran’s *Q* test and *I^2^* statistic. Significant heterogeneity was assumed for *I^2^* > 50% or a *Q*-test *P*-value < 0.05 [31]. We utilized the random-effects model to combine HR from single studies if obvious heterogeneity was observed [32]. Subgroup analyses were conducted to explore potential sources of heterogeneity across studies. In the sensitivity analysis, studies were omitted one by one and the others were analyzed to evaluate the effect of a single study on the summary risk estimates. Publication bias was assessed statistically with Kendall’s tau [33] and Egger’s test [34]. A *P*-value < 0.05 in these tests suggests the presence of publication bias. We utilized STATA (Version 12.0) to perform these analyses. To perform dose–response analyses, information on blood 25(OH)D concentrations were extracted from each study directly if the concentration for each 25(OH)D category was reported as either mean, median, or mid-point. If 25(OH)D concentrations were reported only as intervals, mid-points were calculated. STATA (Version 12.0) was used for the relevant calculation.

## Results

### Literature search and study characteristics

**Figure 1:**
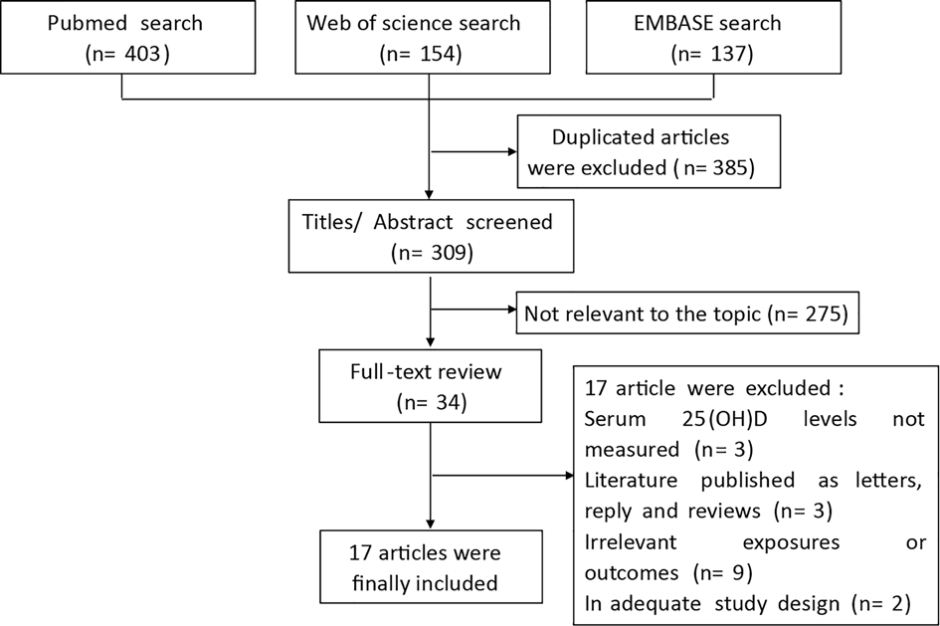
Flow diagram of the systematic literature search for colorectal cancer in PubMed, Web of Science, and EMBASE

We searched PubMed, EMBASE databases, and Web of science from inception to March 1, 2020, for eligible studies on the relationship between vitamin D and mortality of CRC patients. The flow chart of the literature search is presented in Figure 1. After a comprehensive search, a number of 694 literatures were retrieved. About 385 of them were duplicates and discarded. About 309 studies were screened with title and abstract, and 275 of them were excluded as they were not relevant to our topic. The remaining 34 potentially relevant articles underwent a full-text review, of which 17 articles were selected eligible for inclusion in our meta-analysis. One of the 17 articles included two separate cohorts [18], so finally our current meta-analysis includes 18 eligible studies with a total of 17,770 participants.

Basic information concerning the eligible studies is listed in Table 1. A total of 18 prospective cohort studies were included in the meta-analysis of OS (5579 events and 17,770 participants) [18,27,35–49], and 10 of them showed the outcome of CSS (2411 events and 9128 participants) [18,35,36,39–42,45,46]. Nine studies were conducted in the Europe [18,39,40,42,44–46,48], six were performed in US [35,36,38,41,43,49], and the remaining studies were executed in Asia [37,47] and Australia [27]. The sample size of the included studies ranged from 52 to 3818 participants. Five studies investigated stage I-III patients [18,42,47,48], nine studies investigated stage I-IV patients [27,35–37,39–41,45,46] and four studies investigated exclusively stage IV patients [38,43,44,49]. The level of covariate adjustment in the individual studies differed, but most studies adjusted for age, sex, and some indicators relating to tumor spreading. Three studies employed no, or only very limited, adjustment for covariates [42,43,48]. On the basis of the Newcastle–Ottawa scale criteria, 10 articles were classified as high-quality [18,27,35,36,38,39,41,45,46,49], and the remaining studies were classified as moderate-quality [37,40,42–44,47,48].

**Table 1:** Studies reporting on the association of serum 25(OH)D levels (nmol/l) with overall and CRC-specific mortality among CRC patients

Author, year, region	Partic- ipants	Sex	Ages (y) Mean/ Median	Follow-up time (y) Mean/ Median	Stage	Association of 25(OH)D with mortality		Adjustment	Quality
						Overall survival	CRC-specific survival		
						HR (95% CI)	HR (95% CI)		
Ng, 2008, U.S.A. [36]	304	M/F	68	6.5	I-IV	0.52 (0.29–0.93)	0.61 (0.31–1.19)	Age, sex, season, stage, grade, location, BMI, physical activity	9
Ng, 2009, U.S.A. [37]	1017	M/F	66	8	I-IV	0.62 (0.42–0.92)	0.5 (0.26–0.95)	Age, sex, stage, grade, location, year of diagnosis	9
Mezawa, 2010, Asia [38]	257	M/F	65	2.7	I-IV	0.16 (0.04–0.64)	N.R	Age, sex, season, stage, residual tumor, number of lymph nodes with metastasis, time period	8
Ng, 2011, U.S.A. [39]	515	M/F	61	5.1	IV	0.94 (0.72–1.23)	N.R	Age, season, sex, baseline status, treatment arm, BMI, and metastatic sites	9
Fedirko, 2012, 2012 [40]	1202	M/F	62	6	I-IV	0.67 (0.5–0.9)	0.69 (0.5–0.95)	Age, sex, stage, grade, location, smoking, BMI, physical activity, season, diagnosis time, region	9
Tretli, 2012, Europe [41]	52	M/F	59	14	I-IV	0.4 (0.1–1.6)	0.2 (0.04–1.1)	Age, sex, season, time between serum sampling and 25-OHD measurement, and stage	7
Cooney, 2013, U.S.A. [42]	368	M/F	64.8	8.03	I-IV	1.06 (0.64–1.76)	1.01 (0.59–1.74)	Age, stage, race, sex, smoking, month of blood draw, log CRP	9
Zgaga, 2014, Europe [43]	1598	M/F	62	8.9	I-III	0.7 (0.55–0.89)	0.68 (0.5–0.92)	Tumor site, surgery, time between treatment and sampling, season, BMI, physical activity	8
Wesa, 2015, U.S.A. [44]	250	M/F	63	3.4	IV	0.61 (0.38–0.98)	N.R	Albumin and ECOG	8
Facciorusso, 2016, Europe [45]	143	M/F	68	6	IV	0.35 (0.21–0.58)	N.R	Age, sex, serum albumin, INR, CEA, numbers of nodules, max diameter, primary tumor, timing, ECOG	7
Väyrynen, 2016, Europe [46]	117	M/F	67.7	5	I-IV	0.7 (0.29–1.67)	0.99 (0.41–2.43)	Age, sex, tumor location, stage, grade, BMI, season	9
Maalmi, 2017, Europe [47]	2832	M/F	69	4.8	I-IV	0.56 (0.44–0.71)	0.6 (0.45–0.8)	Age, sex, season, BMI, stage, tumor location, tumor detection mode, surgery, chemotherapy, cardiovascular diseases, diabetes, hypertension, smoking, physical activity	9
Yang, 2017, Asia [48]	206	M/F	63	3.75	I-III	1.79 (0.9–3.56)	N.R	Age, sex, smoking, drinking, BMI, diabetes, hypertension, treatment, location, pathological types, stage	7
Zhu, 2019, Oceania [28]	3818	M/F	51.8	20	I-IV	0.87 (0.54–1.4)	N.R	Age, sex, season, vitamin D supplements, marital status, occupation, smoking, alcohol, LTPA, BMI, diabetes, aspirin use	9
Markotic, 2019, Europe [49]	515	M/F	65.8	5.9	I-III	0.81 (0.59–1.09)	N.R	Month of sampling, primary tumor location	7
Yuan, 2019, U.S.A. [50]	1041	M/F	59	5.6	IV	0.66 (0.53–0.83)	N.R	Age, sex, race, ECOG, chemotherapy, treatment arm, BMI, physical activity, season, region	9
*Vaughan-Shaw, 2020, Europe [19]	1687	M/F	61.5	13.3	I-III	0.69 (0.56–0.84)	0.71 (0.55–0.92)	Age, sex, stage, BMI, tumor site, time between definitive treatment, and sampling	9
#Vaughan-Shaw, 2020, Europe [19]	1848	M/F	67.6	3.6	I-III	0.63 (0.44–0.89)	0.62 (0.4–0.95)	Age, sex, stage, BMI, tumor site, time between definitive treatment, and sampling	9

Abbreviations: 25(OH)D, 25-hydroxyvitamin D; BMI, body mass index; CEA, Carcinoembryonic Antigen; CI, confidence interval; CRC, colorectal cancer; CRP, C-reactive protein; ECOG, Eastern Cooperative Oncology Group performance status; HR, hazard ratio; INR, International Normilized Ratio; LTPA, leisure time physical activity. M: male; F: female; N.R: not reported; * Cohort1 studied by Vaughan-Shaw; # Cohort2 studied by Vaughan-Shaw.

### Meta-analysis

**Figure 2:**
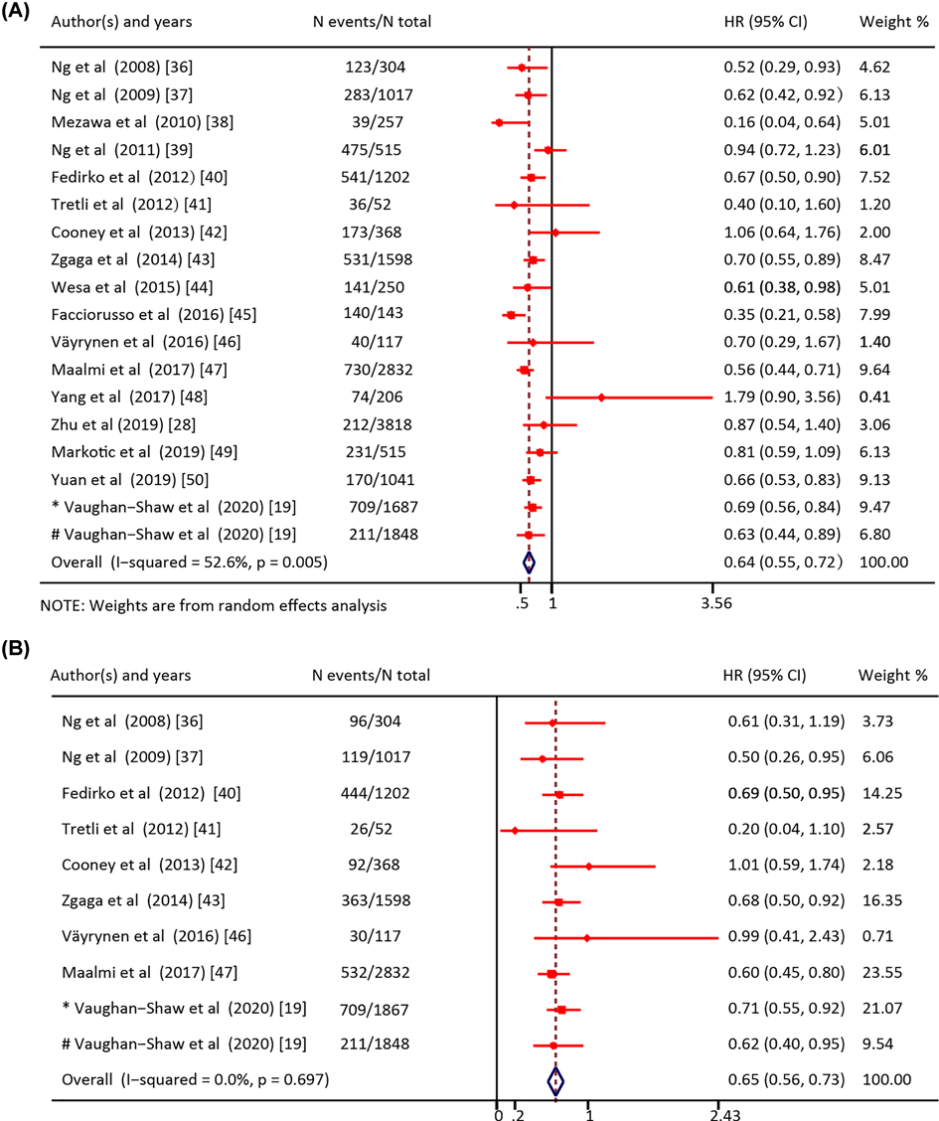
Forest plot for the association of high versus low 25(OH)D levels in patients with colorectal cancer (**A**) Forest plot for the association of high versus low 25(OH)D levels with overall survival in patients with colorectal cancer. (**B**) Forest plot for the association of high versus low 25(OH)D levels with CRC-specific survival in patients with colorectal cancer. HR: Hazard ratio; CI: Confidence interval. * Cohort1 studied by Vaughan-Shaw; # Cohort2 studied by Vaughan-Shaw.

The meta-analysis showed significantly higher overall (HR = 0.64; 95% CI = 0.55–0.72) (Figure 2A) and CRC-specific survival (HR = 0.65; 95% CI = 0.56–0.73) (Figure 2B) in patients with higher circulating 25(OH)D levels compared with those with lower levels. When performing meta-analysis of overall survival, there was a significant but moderate heterogeneity among those included studies (Q (*df* = 17) = 35.9, *P*-value = 0.005; *I*^2^ = 52.6%). However, no significant heterogeneity among the studies was found for CRC-specific survival (Q (*df* = 9) = 6.4, *P*-value = 0.697; *I*^2^ = 0%).

### Sensitivity analysis and publication bias

**Figure 3:**
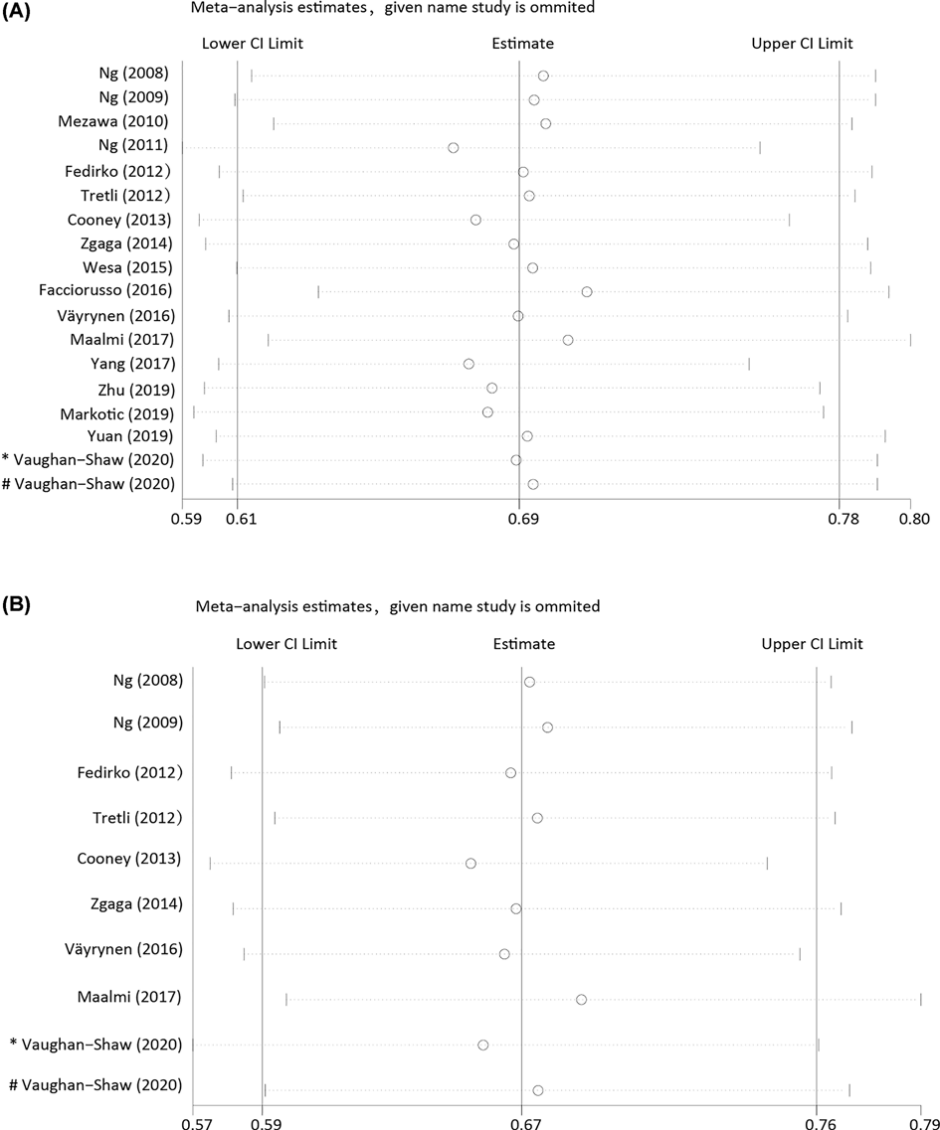
Sensitivity analysis by excluding studies by turns suggested that the pooled HR were not significantly changed by any individual study (**A**) Sensitivity analysis of the association between 25(OH)D and overall survival. (**B**) Sensitivity analysis of the association between 25(OH)D and CRC-specific survival. CI: Confidence interval. * Cohort1 studied by Vaughan-Shaw; # Cohort2 studied by Vaughan-Shaw.

Sensitivity analyses were conducted to examine the stability of the estimates for overall (Figure 3A) and CRC-specific survival (Figure 3B). The sensitivity analysis showed the summary HR was not markedly changed by an individual study, indicating no significant influence of single study on the results. In publication bias analysis, non-significant publication bias was found for either overall (Kendall’s tau = -0.83, *P*=0.43; Egger’s *t* value = -0.48, *P*=0.64) or CRC-specific survival (Kendall’s tau = -0.63, *P*=0.59; Egger’s *t* value = -0.59, *P*=0.57). The funnel plot also suggested that no publication bias exists (Supplementary Figure S1).

### Subgroup analyses

**Figure 4:**
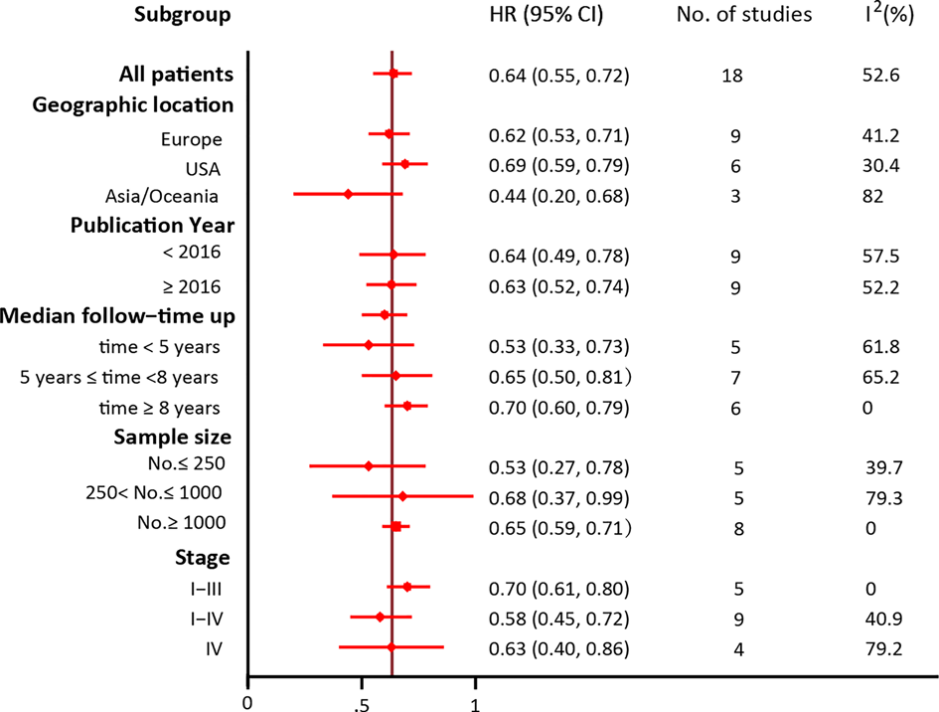
Subgroup analyses of multivariate analysis and HR (95% CI) for overall survival in CRC patients HR: Hazard ratio; CI: Confidence interval

In view of the obvious heterogeneity in overall survival, we conducted a subgroup analysis to detect the source of heterogeneity, which was presented in Figure 4. The included HR for each study was multivariate-adjusted estimates. As our data showed, studies conducted in U.S.A., with median follow-up time ≥ 8 years, a larger sample size, and including stage I-III patients showed a more prominent association between 25(OH)D concentrations and overall survival and the lowest level of heterogeneity. However, the results did not show a difference between subgroups, when the publication year enrolled as a grouping feature. Since no significant heterogeneity was found for CRC-specific survival, we didn’t conduct the subgroup analysis among the studies.

### Dose–response meta-analysis

**Figure 5:**
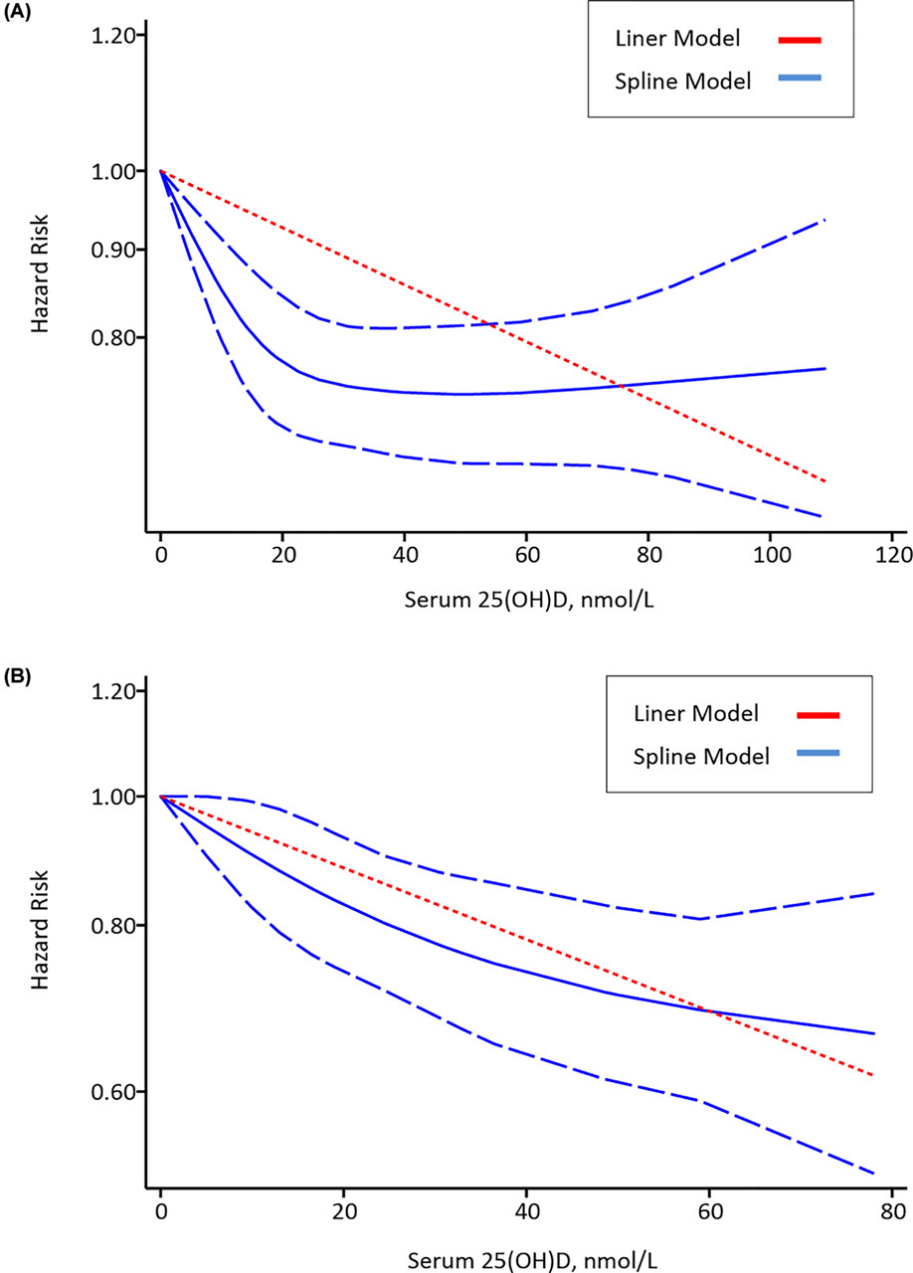
Dose–response relationship between 25(OH)D and risk of all-cause mortality and CRC-specific mortality (**A**) Risk estimates with 95% CI for the association between 25(OH)D and all-cause mortality. (**B**) Risk estimates with 95% CI for the association between 25(OH)D and CRC-specific mortality.

Dose–response analysis indicated a negative correlation between serum 25(OH)D concentration and risk of all-cause mortality or CRC-specific mortality. The liner regression equation showed that the risk of all-cause mortality was reduced by 7% (HR = 0.93; 95% CI: 0.90, 0.95) (Figure 5A), and the risk of CRC-specific mortality was reduced by 12% (HR = 0.88; 95% CI: 0.84, 0.93) (Figure 5B) for each 20 nmol/l increment of 25(OH)D concentration.

## Discussion

The role of circulating 25(OH)D and survival outcomes in CRC patients remains unclear and controversial. This meta-analysis focused on the relationship between 25(OH)D and mortality in CRC, involving 18 studies and 17,770 participants with survival outcomes. The results found higher circulating 25(OH)D concentration was significantly associated with decreased all-cause mortality and CRC-specific mortality. Further dose–response analysis showed that every 20 nmol/l increment of 25(OH)D level was associated with a 7% lower risk of all-cause mortality and a 12% lower risk of CRC-specific mortality. Studies of CRC-specific survival showed good heterogeneity, however, a significant but moderate heterogeneity between studies was found for overall survival. So, we conducted the subgroup analysis, and found that associations were most prominent and heterogeneity lowest in studies that were conducted in the U.S.A., with median follow-up time ≥ 8 years, a larger sample size, and including stage I-III patients.

Numerous evidences supported that circulating 25(OH)D level correlates with the risk and survival of CRC [27,49]. Study showed that 25(OH)D serum level was significantly lower among colorectal cancer patients compared to the healthy control group [50]. In addition, serum 25(OH)D levels among CRC patients with stage IV [49], undergoing surgery and chemotherapy [51], were significantly deficient. According to previous studies, vitamin D has the properties of anti-inflammation [13], anti-oxidative stress [14], reducing cancer cell proliferation [15], and regulating cancer cell differentiation [16]. Furthermore, several inflammatory processes and cytokines, such as TLR4 pathway, interleukin (IL)-6, and IL-17, involved in CRC progression, are regulated by vitamin D [23,52]. In addition, vitamin D could promote the differentiation of colon carcinoma cells by the induction of E-cadherin and the inhibition of β-catenin signaling [53], and might regulate spliceosome and thus, play a role in alternative splicing and possibly microRNA synthesis in colon cancer cells [54]. The above evidences might be an explanation for the protective role of vitamin D in CRC prognosis.

Previous studies reported conflicting results about the 25(OH)D and all-cause mortality and CRC-specific mortality. In several cohort studies, higher 25(OH)D levels were associated with lower total cancer incidence and lower total cancer mortality in colorectal cancer, while there were also studies reported null association [25–27] or U-shaped association [28]. The conflicting findings in the relationship between 25(OH)D and CRC may result from the some factors, such as different populations, various study designs and different confounding factors. The results of our study suggest that 25(OH)D is more likely to be a protective factor during the process of CRC. Therefore, there is still controversy on the role of 25(OH)D in CRC, which need to be elucidated in future researches.

Our study focused on the impacts of high level 25(OH)D on the prognosis of colorectal cancer patients, and found that CRC patients with high circulating 25(OH)D levels have a better prognosis for overall survival and CRC-specific survival. Several systematic reviews or meta-analyses demonstrated the negative association between 25(OH)D and the risk of colorectal cancer [55–57]. Many systematic reviews also showed the effect of 25(OH)D on cancer prognosis [58,59]. There were also some meta-analyses focused on the impact of 25(OH)D on the prognosis of colorectal cancer [29,30,60,61]. However, some of the meta-analyses were only included a limited number of studies [29,30,61]. In addition, quantitative analysis results were not given in some meta-analysis [60]. Therefore, we summarized high-quality studies to perform a detailed systematic and comprehensive quantitative analysis to fill the gap. Given the large sample size in our study, our results provide strong evidence for 25(OH)D levels playing a large role in overall survival and CRC-specific survival of colorectal cancer patients.

The HR (95% CI) for overall and CRC-specific survival in CRC patients from our study was similar to the previous meta-analyses [29,30]. However, our study showed a lower heterogeneity for overall survival than those in the two meta-analyses. The reason might be the larger number studies that we included. For analysis of heterogeneity, the decrease after grouping by a median follow-up time, sample size and staging was obvious. However, in the study conducted by Maalmi et al. stratified analyses by median follow-up time, the results did not show a difference between subgroups [29]. The reason might because they did not discuss the situation when median follow-up time was longer. There is no doubt that studies with a longer median follow-up time and a large sample size will get a more reasonable outcome. As for staging, TNM staging remained a strong tool for establishing prognosis and directing therapy [62]. No significant heterogeneity among the studies was found for CRC-specific survival in our study (*I*^2^ = 0%), which was similar to the results of Maalmi et al. However, the meta-analysis conducted by Xu et al, showed a significant heterogeneity (*I*^2^ = 69%), which might because of less studies they included [30].

Further dose–response analysis in our study, showed that every 20 nmol/l increment of 25(OH)D level was associated with a 7% lower risk of all-cause mortality and a 12% lower risk of CRC-specific mortality. However, the meta-analysis by Maalmi et al. did not show an exact dose-analysis [29]. In addition, the meta-analysis by Xu et al. showed that 25 nmol/l increment in serum 25(OH)D level conferred an all-cause mortality of 0.2 (95%CI -0.01–0.42), and did not conduct dose–response analysis on association between serum 25(OH)D level and CRC-specific mortality [30].

There are potential limitations existing in our study which should be considered. Significant heterogeneity was observed between the studies for overall mortality. However, the heterogeneity only persisted in subgroup that stratifying by the publication year. Some studies included in our meta-analysis tested the circulating 25(OH)D level post diagnosis or post treatment, thus it is difficult to get rid of the possibility of reverse causality. Although all studies adjusted for confounding factors, some potential confounding factors related to 25(OH)D remained residual.

Based on the above results, we can draw the conclusion that higher 25(OH)D level is marginally associated with a risk reduction of all-cause mortality and CRC-specific mortality, indicating 25(OH)D may exert a protective effect in the prognosis of colorectal cancer. Since the majority of the studies were performed in Europe and U.S.A., further prospective studies should be conducted in other regions, with different ethnic origins, to confirm these associations.

## Supplementary Material

Supplementary Figure S1

## Abbreviations

25(OH)D: 25-hydroxyvitamin D
BMI: body mass index
CEA: Carcinoembryonic Antigen
CI: confidence interval
CRC: colorectal cancer
CRP: C-reactive protein
ECOG: Eastern Cooperative Oncology Group performance status
HR: hazard ratio
INR: International Normilized Ratio
LTPA: leisure time physical activity
