# Supplementary material for: Low circulating 25-hydroxyvitamin D level is associated with increased colorectal cancer mortality: a systematic review and dose–response meta-analysis
Source: Biosci Rep. 2020 Jul 29;40(7):BSR20201008. doi: 10.1042/BSR20201008 (PMC7391129; doi:10.1042/BSR20201008)
Supplement: Supplementary Figure S1 [file BSR-2020-1008_supp.pdf]

A

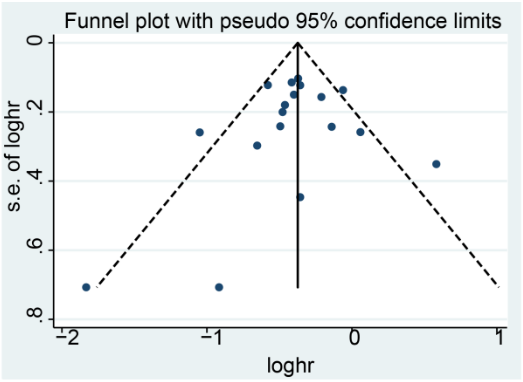

B

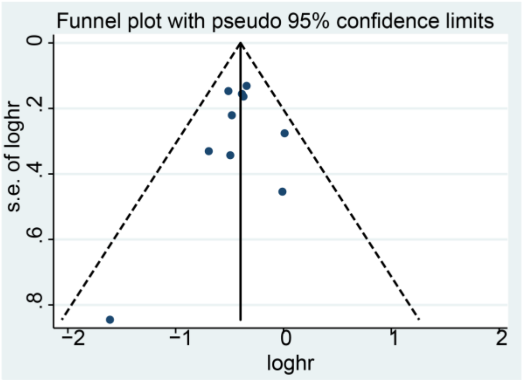

Figure S1. The funnel plots were used to evaluate the publication bias. (A)Funnel plots of the association between 25(OH)D and overall survival. (B) Funnel plots of the association between 25(OH)D and CRC-specific survival.
